# Exploring extracraniofacial anomalies in relation to facial morphology in craniofacial microsomia and microtia

**DOI:** 10.1186/s12887-026-07445-7

**Published:** 2026-08-07

**Authors:** Elsa M. Ronde, Guido A. de Jong, Jitske W. Nolte, Marloes E. L. Nienhuijs, Neil W. Bulstrode, Thomas J. J. Maal, Alfred G. Becking, Corstiaan C. Breugem, and the 3DCFMM research consortium, J. Bolks, D. Dunaway, FA Ebbens, FH Kruisinga, TJJ Maal, EM Ongkosuwito, BJ van Royen, S. Schievano, A. Stadhouder, M. Tjaberinga, EM Ronde, GA de Jong, JW Nolte, MEL Nienhuijs, NW Bulstrode, AG Becking, CC Breugem

**Affiliations:** 1 Department of Plastic, Reconstructive and Hand Surgery, Amsterdam UMC, University of Amsterdam, Amsterdam, the Netherlands; 2 Department of Oral and Maxillofacial Surgery, Amsterdam UMC, University of Amsterdam and ACTA, Academic Centre Dentistry Amsterdam, Amsterdam, the Netherlands; 3 Amsterdam Reproduction and Development Research Institute, Amsterdam, the Netherlands; 4 Amsterdam UMC Expert Center for Cleft, Craniofacial and Airway Disorders, Amsterdam, the Netherlands; 5 Department of Oral and Maxillofacial Surgery, Radboudumc, Nijmegen, the Netherlands; 6 Radboudumc 3D Lab, Radboudumc, Nijmegen, the Netherlands; 7 Department of Plastic Surgery, Great Ormond Street Hospital, London, UK; 8 UCL Great Ormond Street Institute of Child Health, London, UK

**Keywords:** Craniofacial microsomia, Stereophotogrammetry, Vertebral anomalies, Cardiac anomalies, Renal anomalies, Breathing difficulties

## Abstract

**Background:**

Craniofacial microsomia (CFM) is associated with extracraniofacial anomalies, obstructive breathing, and feeding difficulties. This study aimed to explore whether patients with extracraniofacial anomalies, obstructive breathing, or feeding difficulties can be distinguished from patients without these anomalies or difficulties, based on their facial morphology.

**Methods:**

This cross-sectional study used three-dimensional photographs and asymmetry index (ASI), facial signature (FS) scores, and principal components (PC) derived from FS scores to measure facial morphology. Univariate analyses were performed to assess significant differences in ASI, FS, and PC scores, as well as patient characteristics between patients with extracraniofacial anomalies, or feeding and/or breathing difficulties, and patients without these anomalies or difficulties. Multivariate logistic regression analyses were performed for both outcomes.

**Results:**

Extracraniofacial anomalies were found in 84 (47%) out of 179 patients. In the multivariate analysis, the number of associated groups of craniofacial anomalies (OR 2.62 [95% CI 1.72–4.15]) and higher PC30 scores, depicting increasingly negative FS scores around the chin, maxilla, and inferior orbital rims (OR 1.06 [95% CI 1.01–1.11]) were significant independent predictors of extracraniofacial anomalies. For feeding and/or breathing difficulties, independent predictors were clefting (OR 6.45 [95% CI 2.72–16.05]), anomalies of the respiratory or gastrointestinal tracts (OR 7.63 [95% CI 2.33–28.06]), and lower PC34 scores (OR 0.91 [95% CI 0.84–0.97]), corresponding to increasingly positive FS scores of the inferior orbital rims and increasingly negative FS scores of the forehead.

**Conclusions:**

Patients with extracraniofacial anomalies and patients with breathing or feeding difficulties had more severe facial phenotypes. Extracraniofacial anomalies were significantly and independently associated with morphological patterns possibly corresponding to bilateral orbital involvement and chin retrusion, as well as other associated craniofacial anomalies.

**Supplementary Information:**

The online version contains supplementary material available at https://doi.org/10.1186/s12887-026-07445-7.

## Introduction

Craniofacial microsomia (CFM) is a congenital condition involving underdevelopment or hypoplasia of craniofacial structures. Next to a varying degree of hypoplasia, patients with CFM frequently have various other anomalies, such as macrostomia and facial skin tags, as well as anomalies affecting other organ systems and skeletal structures [1].

Extracraniofacial anomalies have been reported to occur in approximately 50% of patients with CFM [2]. Cardiac and vertebral structures are reported to be affected most frequently, leading to anomalies including atrial or ventricular septal defects, as well as split or fused vertebrae (i.e. hemivertebrae or block vertebrae) [2, 3]. Screening patients for these anomalies involves echocardiography and radiographic spinal imaging. Rarer anomalies affecting the respiratory or gastrointestinal systems, such as tracheomalacia and esophageal atresia, may cause significant breathing and feeding difficulties. These anomalies, the often concurrent mandibular hypoplasia, as well as potential oral clefting and macrostomia, make screening for obstructive breathing and monitoring feeding in patients with CFM crucial.

Clinicians have voiced a desire to tailor screening and diagnostics according to the severity of CFM [4]. Previous reports have suggested that obstructive breathing, feeding difficulties, and extracraniofacial anomalies occur more frequently in patients with more severe mandibular hypoplasia according to the Pruzansky-Kaban classification [2, 5, 6]. However, classifying the mandible requires radiographic imaging and is therefore usually not performed in the first diagnostic phase [4].

Digital stereophotogrammetry, also known as three-dimensional (3D) photography, can distinguish patients with CFM from individuals without craniofacial conditions and quantitatively assess the severity of facial involvement [7]. This study aimed to assess whether patients with extracraniofacial anomalies, obstructive breathing or feeding difficulties can be distinguished from patients without these anomalies or difficulties, according to their facial morphology, as measured using 3D photography.

## Methods

This was a multicenter study at Amsterdam UMC, Amsterdam, the Netherlands, Radboudumc, Nijmegen, the Netherlands, and the Great Ormond Street Hospital, London, United Kingdom. Patients with prior 3D photographs were included if they had CFM according to the International Consortium for Health Outcomes Measurement criteria [8], or isolated microtia. Goldenhar syndrome was also considered CFM [8]. Patients were excluded if they had a confirmed other syndrome (e.g. Treacher Collins), if the quality of available 3D photographs was poor or if they had undergone significant craniofacial surgery such as mandibular surgery prior to 3D photography.

Patient data was collected from electronic data files. The collected data included patient characteristics, OMENS or Phenotypic Assessment Tool for Craniofacial Microsomia classification scores, any craniofacial anomalies as reported previously [9], as well as any extracraniofacial anomalies. With regards to extracraniofacial anomalies, data was specifically collected on cardiac, vertebral, urogenital, and limb anomalies. Any other extracraniofacial anomalies were also noted and grouped into respiratory tract anomalies, gastrointestinal tract anomalies, as well as other extracraniofacial anomalies for data analysis. Data on a history of obstructive breathing and feeding difficulties was also collected. Feeding difficulties were considered present if modifications had been made to food consistency (e.g., mincing solid foods) or the delivery method (e.g., tube feeding). Obstructive breathing was defined as any breathing difficulties diagnosed with polysomnography or requiring medical or surgical intervention. Patients with asthma were not considered to have CFM-related obstructive breathing. Anomalies or difficulties were considered absent if these were not mentioned in data records.

This study was exempted from full institutional board review, and informed consent was obtained from patients and/or legal guardians. The Strengthening the Reporting of Observational studies in Epidemiology (STROBE) checklist (Additional File 1) were followed [10].

### 3D photography and assessment of facial morphology – asymmetry index, facial signatures and principal component scores

3D photographs were standardized using 3DMedX version 1.2.28.1 (Radboudumc 3D Lab, Nijmegen, the Netherlands, https://www.3dmedx.nl/) and the integrated MeshMonk toolbox [11]. This involved initial manual landmarking for rough alignment and then both rigid and non-rigid registration to create face models consisting of 7160 bilaterally symmetrical data points [11].

Facial morphology was assessed using asymmetry index (ASI) and facial signature (FS) scores. ASI scores reflect the degree of asymmetry at each data point within an individual face model. These were derived for all unilaterally affected patients by measuring the distance between corresponding data points after superimposition of mirrored and scaled face models using a Klingenberg protocol [12, 13]. FS scores were calculated for all patients to reflect the degree of deviation of each point from an age- and sex-adjusted average face according to the protocol and workflow of Matthews et al. [14]. Briefly, an average face was defined according to publicly available growth models of the face. Then, each face model was aligned and superimposed to the average face, and finally, z scores (i.e., normalized standard deviations) were calculated for point-wise displacements using the average and standard deviation data of the growth models [14]. Both ASI and FS scores were generated for the full face as well as subdivisions of the upper half of the face, the lower half of the face, and the midface, and were summarized using root mean square in an automated workflow in 3DMedX. The subdivisions were selected from a previous hierarchical spectral clustering analysis of facial shape according to clinical relevance in patients with CFM [15].

A model of nine principal components (PCs) was previously validated to distinguish patients with CFM from individuals without craniofacial conditions through a logistic regression of PC scores [7] (Additional File 2). PCs are composite variables that were generated by applying a principal components analysis to the raw FS scores of a matched control population and transforming patient FS scores into the PC space. The regression model represents nine distinct patterns of facial shape associated with CFM, and these nine PCs were included in the analyses of this study as an additional measure for facial morphology.

### Statistical analyses

Primary univariate analyses were performed to assess any significant differences in patient characteristics, ASI, FS, and PC scores, and extracraniofacial anomalies and feeding or breathing difficulties, between patients with and without these anomalies or difficulties. The Mann Whitney U test, Student’s t-test, Pearson’s chi-squared test or Fisher’s exact test were used according to data type, distribution, and the number of observations. Feeding or breathing difficulties were combined into one binary variable: patients without feeding or breathing difficulties versus patients with feeding and/or breathing difficulties due to the number of observations. Subgroup univariate analyses were also performed for patients under the age of 2 years at the time of 3D imaging.

A multivariate logistic regression analysis was performed to identify significant predictors of extracraniofacial anomalies with the following input variables: bilateral CFM, isolated microtia, the number of groups of craniofacial anomalies (grouped as orbital anomalies, skin adnexa-related anomalies, clefting, and aural atresia), and principal components 13, 14, 15, 19, 20, 23, 30, 33 and 34. These variables were chosen because of the hypothesis that extracraniofacial anomalies occur more frequently in bilaterally affected patients, patients with more severe facial involvement, and patients with more associated craniofacial anomalies and less frequently in patients with isolated microtia. The groups of craniofacial anomalies were selected for being reliably extractable from patient files and images without radiographic imaging, and were used as a composite variable to reduce the number of predictors in the regression..

Another multivariate logistic regression analysis was performed to identify significant predictors of feeding and/or breathing difficulties with the following input variables: bilateral CFM, clefting, respiratory and/or gastrointestinal system anomalies, and principal components 13, 14, 15, 19, 20, 23, 30, 33 and 34. These variables were chosen since we hypothesized that feeding and/or breathing difficulties were more likely to occur in bilaterally affected patients, patients with clefting, respiratory system and/or gastrointestinal system anomalies, patients with more severe facial involvement, and patients with more associated craniofacial anomalies. Isolated microtia could not be used as a predictor in the multivariate analysis due to insufficient observations of breathing and/or feeding difficulties in this group of patients.

Analyses were conducted using R version 4.2.1 (R Core Team, Vienna, Austria) via the RStudio interface [16]. Data was presented as means with standard deviations, medians with an interquartile range or as the number and proportion (%) of observations according to data type and distribution. Regression results are presented as odds ratios with 95% confidence intervals. A p-value < 0.05 was considered statistically significant. The Benjamini–Hochberg method was used to adjust p values for the subgroup analyses of patients under 2 years of age [17].The morphological patterns represented by the PCs were visualized using the original FS scores for more intuitive interpretation.

## Results

In this study, 179 patients with CFM or microtia were analyzed for facial morphology, craniofacial anomalies and extracraniofacial anomalies. The median age of included patients was 9 years (interquartile range 5–14 years). Out of these patients, 11% had bilateral CFM and 15% had isolated microtia according to their OMENS scores. Patients most frequently had an orbit score of 0, a mandible score of 2 A or 2B, lobule type microtia, no facial nerve weakness and minimal soft tissue deficiency, respectively, according to the OMENS classification (Table 1). Patients most frequently had an associated craniofacial anomaly in two of the following groups: ocular anomalies, skin-adnexa related anomalies, clefting and aural atresia.

**Table 1:** Patient characteristics

**Variable**^**1**^	Total *n* = 179
Patient characteristics	
Age at 3D imaging	9 (5–14), yrs
Sex, male	98 (55%)
Bilateral CFM^2^	19 (11%)
Isolated microtia^3^	15 (8.4%)
OMENS	
Orbit, normal size and position	78 (44%)
Mandible, Pruzansky-Kaban 2 A or 2B	57 (32%)
Ear, lobule type	72 (40%)
Nerve, no facial nerve weakness	65 (26%)
Soft tissue, minimal deficiency	86 (48%)
Craniofacial anomalies	
Ocular anomalies	44 (25%)
Skin adnexa-related anomalies	101 (56%)
Nerve weakness	94 (53%)
Clefting	57 (32%)
Aural atresia	146 (82%)
Middle ear anomalies	75 (42%)
Inner ear anomalies	33 (18%)
Extracraniofacial anomalies	84 (47%)
Cardiac anomalies	41 (23%)
Vertebral anomalies	39 (22%)
Limb anomalies	22 (12%)
Urogenital system anomalies	19 (11%)
Gastrointestinal system anomalies	14 (7.8%)
Respiratory system anomalies	10 (5.6%)
Other system anomalies	12 (6.7%)
Feeding and/or breathing difficulties	51 (28%)
Feeding difficulties	35 (20%)
Breathing difficulties	29 (16%)

*Abbreviation: CFM, craniofacial microsomia; OMENS, Orbit, Mandible, Ear, Nerve, and Soft tissue classification*

^1^Median (IQR) or Number (%)

^2^According to the OMENS classification

^3^No orbital, mandibular, nerve or soft tissue involvement according to OMENS

Extracraniofacial anomalies were described in 84 (47%) patients. Cardiac anomalies were reported most frequently in 23% of patients, followed by vertebral anomalies and limb anomalies. Septal defects of the heart, scoliosis, and fusion or segmentation defects of the vertebrae, as well as lower limb length discrepancies were reported most frequently for these groups of anomalies, respectively (Additional File 3). Furthermore, feeding and/or breathing difficulties were noted in 28% of patients (Table 1).

Both ASI and FS scores of the full face (region 1) were significantly higher in patients with at least one extracraniofacial anomaly or a history of feeding and/or breathing difficulties, compared with those without extracraniofacial anomalies or breathing and/or feeding difficulties (Table 2). Significant differences were also seen in several subregions, such as the midface (region 2), cheeks (region 8), and orbits (region 11) (Table 2). When assessing extracraniofacial anomalies separately, significantly higher ASI and FS scores were mostly found in patients with cardiac anomalies compared with those without (Additional Files 4 and 5).

**Table 2:** Asymmetry index and facial signature scores for patients with and without extracraniofacial anomalies, or feeding and/or breathing difficulties for each facial region

Regions^1^			1	2	3	4	5	6	7	8	9	10	11
												
**ASI**	*Any ECFA*	**Yes**, *n* = 76	1.75**	1.07**	1.81**	1.07**	0.87*	1.91**	1.28*	1.61**	2.07*	1.10◦	1.34**
			[1.13–2.33]	[0.70–1.54]	[1.15–2.44]	[0.69–1.73]	[0.64–1.31]	[1.10–2.87]	[0.94–1.67]	[1.02–2.43]	[1.17–3.31]	[0.74–1.61]	[0.91–1.63]
		**No**, *n* = 84	1.26	0.86	1.31	0.83	0.71	1.41	1.12	1.17	1.58	0.97	1.07
			[0.92–1.77]	[0.64–1.09]	[0.94–1.88]	[0.61–1.17]	[0.55–0.97]	[0.86–2.29]	[0.74–1.43]	[0.74–1.83]	[0.92–2.32]	[0.63–1.33]	[0.76–1.42]
	*History of FBD*	**Yes**, *n* = 41	1.90**	1.26***	2.01**	1.47***	1.05*	2.30*	1.31*	1.94*	2.33*	1.13◦	1.32
			[1.23–2.21]	[0.86–1.74]	[1.19–2.32]	[0.85–1.84]	[0.67–1.62]	[1.30–2.79]	[1.03–1.70]	[1.09–2.47]	[1.28–3.12]	[0.75–1.72]	[1.00-1.69]
		**No**, *n* = 119	1.29	0.88	1.35	0.85	0.75	1.49	1.15	1.26	1.67	1.05	1.17
			[0.95–1.87]	[0.64–1.13]	[0.98–2.02]	[0.62–1.20]	[0.55–1.04]	[0.94–2.36]	[0.81–1.44]	[0.81–1.94]	[0.98–2.55]	[0.62–1.43]	[0.81–1.48]
**FS**	*Any ECFA*	**Yes**, *n* = 84	1.47*	1.31*	1.53◦	1.19	1.20**	1.56*	1.32	1.31**	1.60	1.21	1.29*
			[1.18–1.84]	[0.95–1.71]	[1.15–1.86]	[0.88–1.66]	[0.94–1.70]	[1.13–2.03]	[1.10–1.60]	[0.98–1.86]	[1.17–2.25]	[0.93–1.61]	[1.01–1.56]
		**No**, *n* = 95	1.31	1.11	1.34	1.14	1.01	1.32	1.23	1.08	1.40	1.05	1.15
			[1.10–1.56]	[0.91–1.36]	[1.11–1.63]	[0.87–1.49]	[0.86–1.28]	[1.08–1.79]	[0.93–1.54]	[0.86–1.48]	[1.13–1.84]	[0.81–1.61]	[0.90–1.42]
	*History of FBD*	**Yes**, *n* = 51	1.61***	1.38***	1.64***	1.44***	1.32**	1.81***	1.38*	1.44**	1.74***	1.19	1.39**
			[1.24–2.03]	[1.05–1.83]	[1.31–1.95]	[1.05–1.82]	[1.01–1.77]	[1.32–2.10]	[1.16–1.66]	[1.06–1.88]	[1.39–2.44]	[0.96–1.72]	[1.14–1.58]
		**No**, *n* = 128	1.31	1.08	1.33	1.07	1.02	1.32	1.22	1.11	1.37	1.16	1.17
			[1.09–1.51]	[0.90–1.38]	[1.09–1.57]	[0.80–1.42]	[0.84–1.34]	[1.05–1.79]	[0.93–1.54]	[0.88–1.53]	[1.08–1.87]	[0.80–1.59]	[0.93–1.42]
***Abbreviations***: *ASI*,* asymmetry index; FS*,* facial signature; ECFA*,* extracraniofacial anomalies; FBD*,* feeding and/or breathing difficulties*													
^1^Median (interquartile range) root mean square													

***p < 0.001, **p < 0.01, *p < 0.05, ◦ *p* ≤ 0.1, Mann Whitney U test

**Fig. 1:**
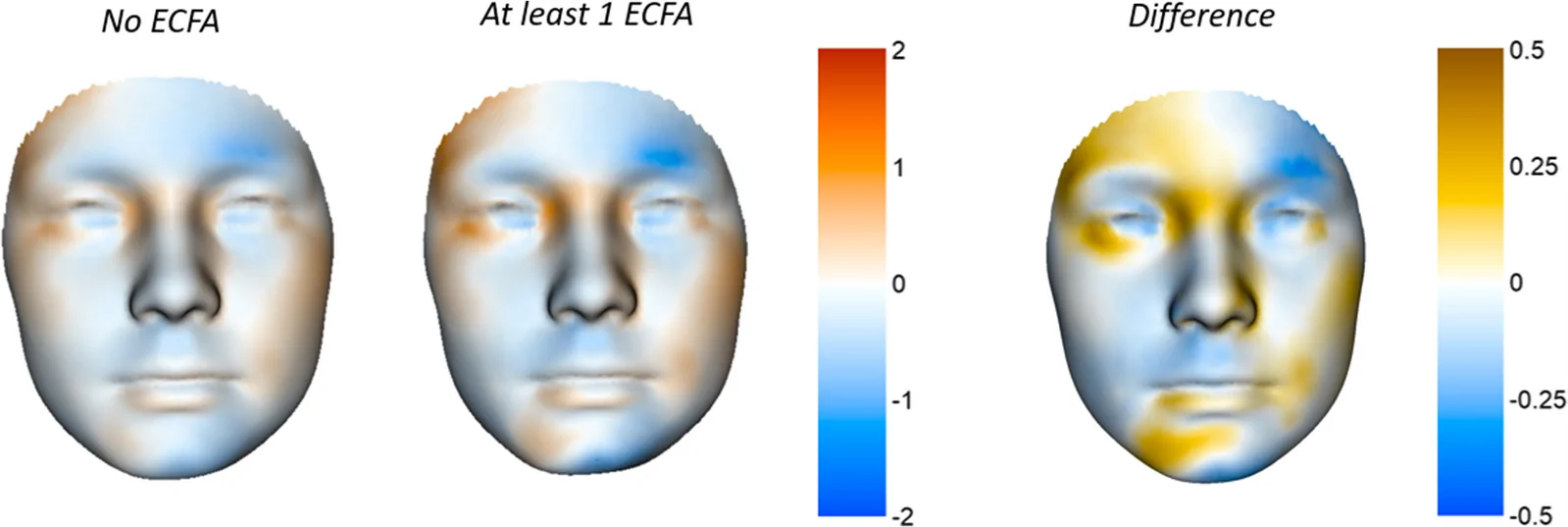
A mean patient without (left) and with at least one extracraniofacial anomaly (center) visualized using principal components 20, 23, 30 and 34, according to the univariate analysis. The corresponding facial signature scores are projected as colors onto the image, where orange signifies positive facial signature scores and blue negative facial signature scores. The difference in facial morphology between these two groups is visualized on the right with a smaller range (−0,5 to 0.5) to showcase subtle differences where yellow signifies positive facial signature scores and blue negative facial signature scores

**Fig. 2:**
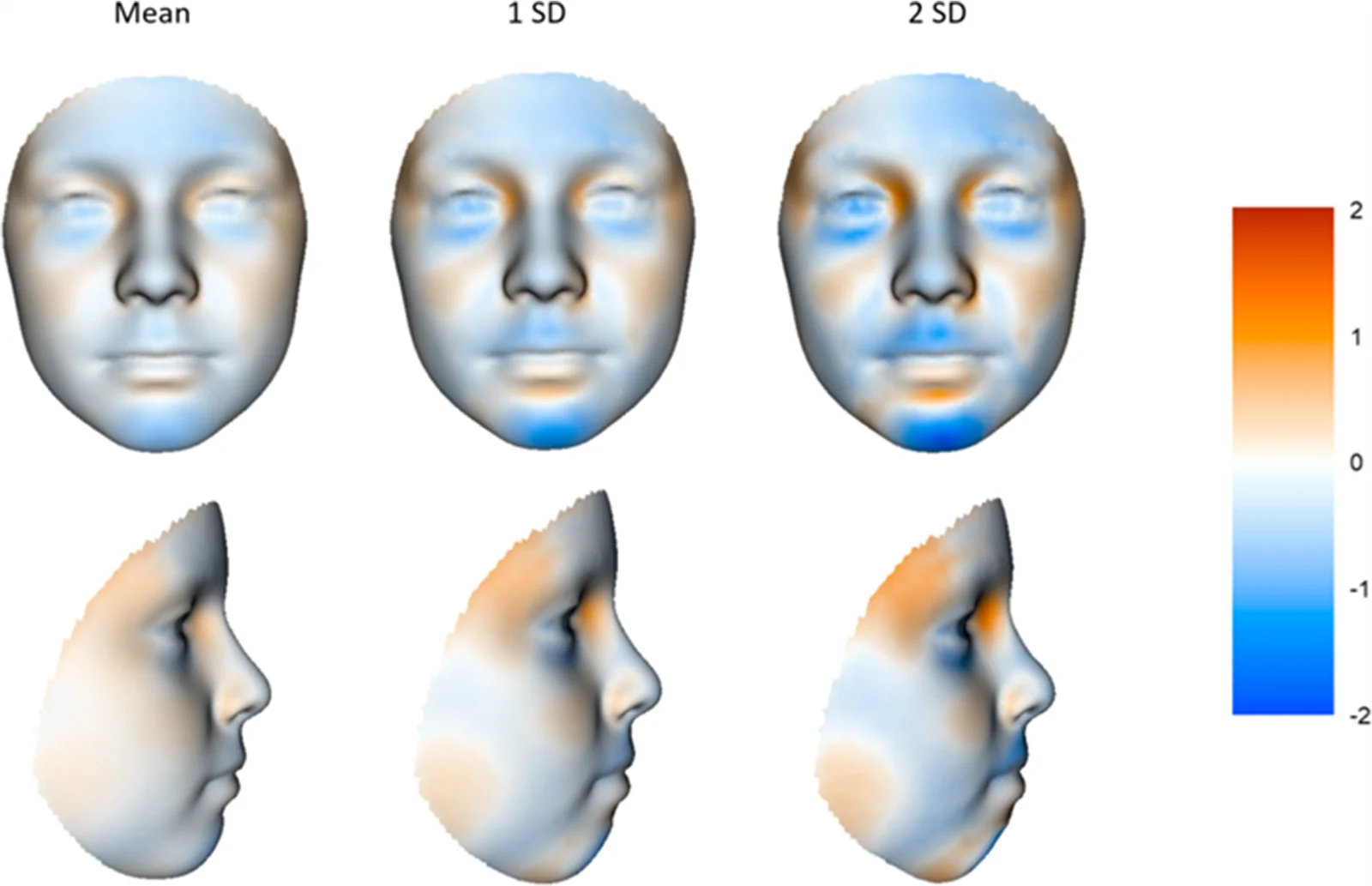
A visual representation of the morphological pattern of principal component 30, illustrating progressive chin retrusion with increasing principal component scores. The top row shows the pattern on a template face, and the bottom row shows the morphological change with increasing PC scores. The overlaid colors represent the corresponding facial signature scores, where orange signifies positive facial signature scores and blue negative facial signature scores

In the univariate analysis patients with extracraniofacial anomalies were more likely to have concurrent craniofacial anomalies in several groups compared with patients without extracraniofacial anomalies. They also had lower scores in PCs 20, 23 and 34, as well as higher PC30 scores (Table 3). When visualizing these components, patients with extracraniofacial anomalies had more chin deviation, as well as more orbital involvement (Fig. 1). In the multivariate analysis, the number of groups of craniofacial anomalies (OR 2.62 [95% CI 1.72–4.15]), as well as PC30 (OR 1.06 [95% CI 1.01–1.11]) remained significant independent predictors of a history of extracraniofacial anomalies (Table 3). The morphological pattern associated with increasing PC30 scores depicted increasingly negative facial signature scores around the chin, upper lip, and the inferior orbital rims in a relatively symmetrical manner (Fig. 2).

**Table 3:** Results of univariate and regression analyses for a history of extracraniofacial anomalies

Type of variable	History of extracraniofacial anomalies^1^			Multivariate regression	
	**No,** n = 95	**Yes,** n = 84	*p-value*^*2*^	**OR (95% CI)**	*p-value*
**Patient characteristics**					
Bilateral CFM^3^	11 (12%)	8 (9.5%)	0.7	0.37 (0.11–1.13)	0.089
Isolated microtia^4^	13 (14%)	2 (2.4%)	**0.006**	0.43 (0.06–1.87)	0.306
**No. of groups of craniofacial anomalies**^**5**^			** < 0.001**	2.62 (1.72–4.15)	** < 0.001**
0	8 (8.4%)	0 (0%)			
1	39 (41%)	15 (18%)			
2	38 (40%)	32 (38%)			
3	7 (7.4%)	27 (32%)			
4	3 (3.2%)	10 (12%)			
**Principal components**					
PC13	−5.13 (11.64)	−4.81 (12.62)	0.9	1.01 (0.98–1.05)	0.338
PC14	−7.54 (15.98)	−9.35 (19.83)	0.5	1.00 (0.97–1.02)	0.779
PC15	7.48 (15.71)	8.00 (19.70)	0.8	0.98 (0.95–1.01)	0.135
PC19	−1.40 (9.97)	−3.31 (15.57)	0.3	0.99 (0.95–1.02)	0.511
PC20	−14.57 (14.58)	−21.86 (22.04)	**0.009**	1.02 (0.99–1.05)	0.183
PC23	−6.18 (8.83)	−9.81 (11.34)	**0.017**	0.96 (0.91–1.00)	0.082
PC30	8.25 (10.98)	13.49 (11.87)	**0.002**	1.06 (1.01–1.11)	**0.013**
PC33	2.03 (5.92)	2.16 (7.19)	0.9	0.99 (0.93–1.05)	0.668
PC34	−0.73 (5.68)	−2.42 (6.20)	**0.059**	0.95 (0.89–1.01)	0.085

*Abbreviations*: *CFM* craniofacial macrosomia, *PC* principal component, *OMENS* Orbit, Mandible, Ear, Nerve, and Soft tissue classification

^1^Mean (SD) or Number (%)

^2^Two Sample t-test; Pearson’s Chi-squared test; Fisher’s exact test

^3^According to the OMENS classification

^4^No orbital, mandibular, nerve or soft tissue involvement according to OMENS

^5^Orbital anomalies, skin adnexa-related anomalies, clefting, aural atresia

**Fig. 3:**
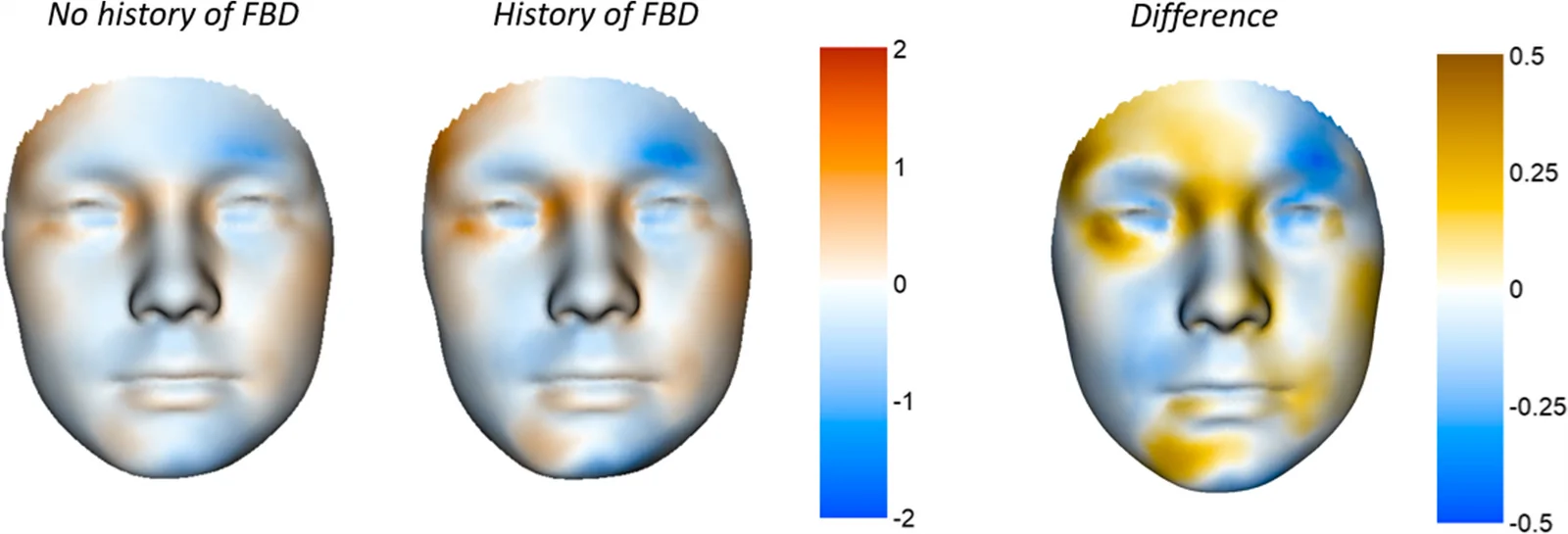
A mean patient without (left) and with feeding and/or breathing difficulties (center), visualized using principal components 20, 23, 30, 33 and 34 according to the univariate analysis. The corresponding facial signature scores are projected as colors onto the image, where orange signifies positive scores and blue negative scores. The difference in facial morphology between these two groups is visualized on the right with a smaller range (−0.5 to 0.5) to showcase subtle differences where yellow signifies positive facial signature scores and blue negative facial signature scores

**Fig. 4:**
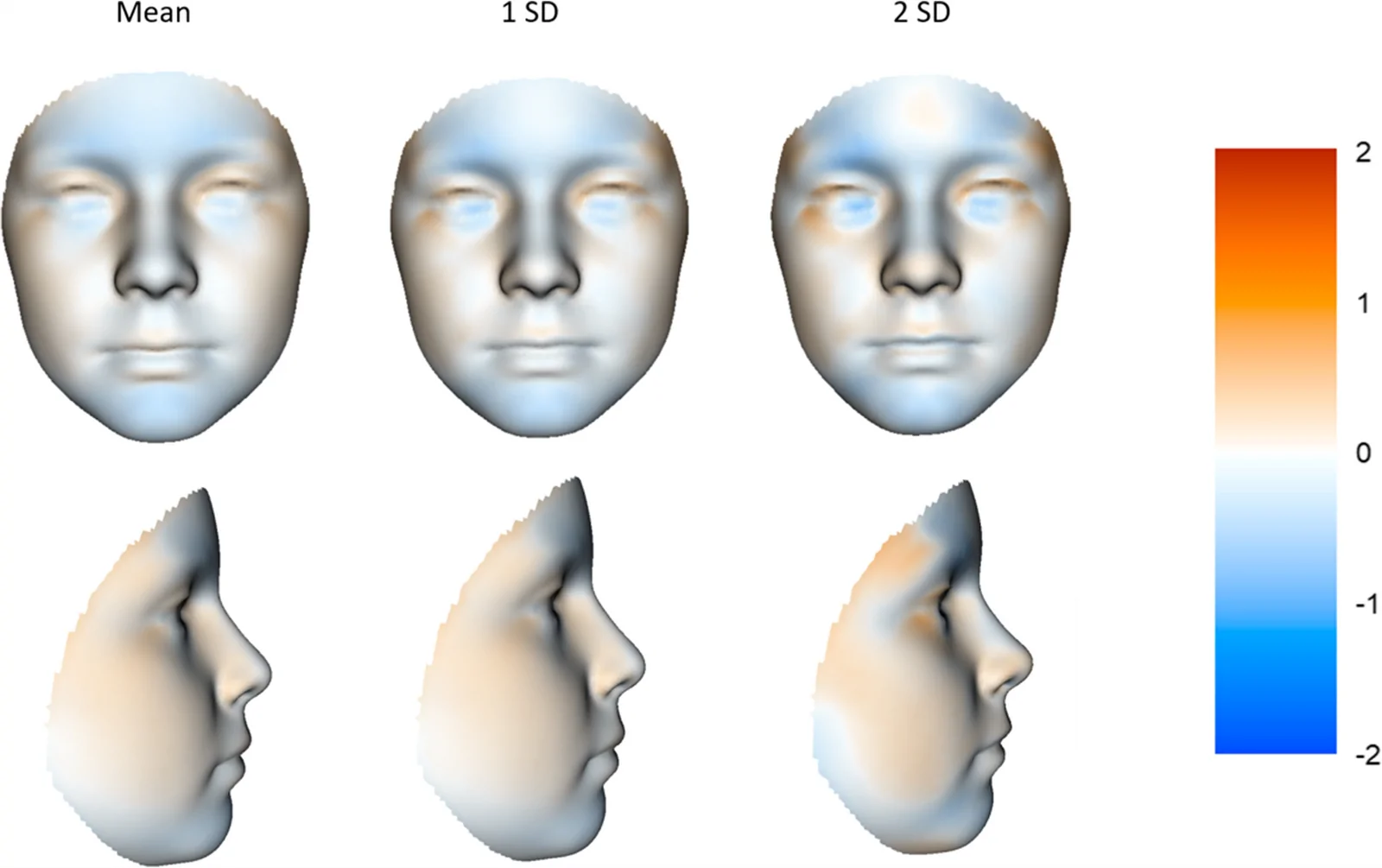
A visual representation of the morphological pattern of principal component 34. The overlaid colors represent the corresponding facial signature scores, where orange signifies positive facial signature scores and blue negative facial signature scores

In the univariate analyses, patients with feeding and/or breathing difficulties were more frequently bilaterally affected according to OMENS scores, were more likely to have a history of clefting, and respiratory, and/or gastrointestinal tract anomalies. They were also more likely to have lower scores in PCs 20, 23 and 34, and higher scores in PCs 30 and 33 (Table 4). Visually, these components corresponded to more chin deviation and increasing orbital, frontal, and glabellar involvement in individuals with feeding and/or breathing difficulties (Fig. 3). In the multivariate analysis, clefting (OR 6.45 [95% CI 2.72–16.05]), respiratory and/or gastrointestinal tract anomalies (OR 7.63 [95%CI 2.33–28.06]), as well as lower scores in PC34 (OR 0.91 [95%CI 0.84–0.97]) remained significant independent predictors of feeding and/or breathing difficulties (Table 4). The morphological pattern associated with decreasing PC34 scores involved increasingly positive facial signature scores of the inferior orbital rims, as well as increasingly negative facial signature scores of the forehead (Fig. 4).

**Table 4:** Results of univariate and regression analyses for a history of breathing and/or feeding difficulties

Type of variable	History of breathing and/or feeding difficulties^1^			Multivariate regression	
	**No,** n = 128	**Yes,** n = 51	*p-value*^*2*^	**OR (95% CI)**	*p-value*
Patient characteristics					
Bilateral CFM^3^	9 (7.0%)	10 (20%)	**0.014**	2.61 (0.73–9.27)	0.136
**Craniofacial anomalies**					
Clefting, yes	24 (19%)	33 (65%)	** < 0.001**	6.45 (2.72–16.05)	** < 0.001**
**Extracraniofacial anomalies**					
Respiratory and/or gastrointestinal system anomalies, yes	6 (4.7%)	16 (31%)	** < 0.001**	7.63 (2.33–28.06)	**0.001**
**Principal components**					
PC13	−4.42 (12.19)	−6.40 (11.79)	0.3	0.97 (0.94–1.01)	0.121
PC14	−8.62 (16.52)	−7.84 (21.01)	0.8	1.00 (0.97–1.03)	0.871
PC15	6.27 (15.24)	11.37 (22.34)	0.081	0.98 (0.95–1.02)	0.359
PC19	−2.61 (10.99)	−1.51 (16.88)	0.6	1.02 (0.98–1.07)	0.253
PC20	−15.54 (16.33)	−24.12 (22.85)	**0.005**	1.00 (0.96–1.03)	0.874
PC23	−6.69 (9.45)	−10.88 (11.50)	**0.013**	0.98 (0.92–1.03)	0.442
PC30	9.48 (10.28)	13.78 (14.26)	**0.026**	0.99 (0.94–1.04)	0.726
PC33	1.55 (6.05)	3.44 (7.49)	**0.080**	1.04 (0.97–1.11)	0.271
PC34	−0.62 (5.63)	−3.79 (6.25)	**0.001**	0.91 (0.84–0.97)	**0.008**

*Abbreviations*: *FBD* feeding and/or breathing difficulties, *OR* odds ratio, *CI* confidence interval, *CFM* craniofacial macrosomia, *PC* principal component, *OMENS* Orbit, Mandible, Ear, Nerve, and Soft tissue classification

^1^Mean (SD) or Number (%)

^2^Two Sample t-test; Pearson’s Chi-squared test; Fisher’s exact test

^3^According to the OMENS classification

In the subgroup of patients under 2 years of age, significantly higher FS scores were found in patients with feeding and/or breathing difficulties in the full face (region 1) and several subregions compared to those without these difficulties (Additional File 6). No statistically significant differences were seen in ASI or FS scores for patients with or without extracraniofacial anomalies (Additional File 6). Differences in PC scores were not statistically significant for either outcome (Additional File 7). Visually, subgroup patients with extracraniofacial anomalies had more mandibular and orbital involvement, compared to those without, while minimal differences involving the orbits were seen in patients with feeding and/or breathing difficulties (Additional File 8).

## Discussion

This study assessed the relationship between the severity of facial involvement and extracraniofacial anomalies, as well as breathing and feeding difficulties in patients with CFM.

Extracraniofacial anomalies were significantly and independently associated with a facial phenotype characterized by lower facial signature scores around the chin, maxilla, and bilateral inferior orbital rims. Previous studies found correlations between extracraniofacial anomalies and higher total OMENS scores [18] or higher Pruzansky-Kaban scores [2]. Extracraniofacial anomalies have also been reported more frequently in bilaterally affected patients [1, 2]. In the current study, clinically bilateral CFM was not associated with extracraniofacial anomalies. This discrepancy could be due to a difference in definition of bilateral CFM; in the present study, bilateral CFM was defined by any positive OMENS subscale score on both sides of the face. In Renkema et al., the definition is not explicitly mentioned [2], while in Caron et al., only the Pruzansky-Kaban score was used to determine laterality [1]. Our results seem to support the conclusion that bilateral hypoplasia of at least the mandible is associated with extracraniofacial anomalies, considering that PC30 shows a clearer pattern of chin retrusion as opposed to chin deviation.

In contrast to previous studies, the current results may also indicate an association between extracraniofacial anomalies and bilateral orbital involvement, possibly corresponding to deeper set orbits or decreased orbital size. Decreased orbital size has been reported as a characteristic of orbital morphology on the affected side of unilateral CFM patients in a previous study [19]. In the current study, PC30 also illustrated higher FS scores medially to the orbits, perhaps signifying a degree of hypertelorism or telecanthus. Other previous studies reporting on associations with extracraniofacial anomalies have used the orbit subscale scores of the OMENS [2, 18], which may not be appropriate for reliably assessing orbital involvement [4, 7, 20, 21], especially considering that it uses the contralateral side as comparison. It also does not consider the possibility of lateral displacement. Further skeletal imaging analyses on orbital dimensions in patients with CFM accompanied by 3D photography seem warranted to further investigate the skeletal changes associated with the orbital morphology in PC30.

Extracraniofacial anomalies were also significantly and independently associated with other craniofacial anomalies. Notably, 44% of patients with extracraniofacial anomalies had concurrent ocular anomalies, skin adnexa-related anomalies, clefting, and aural atresia, or anomalies in at least three of the aforementioned groups, compared with just 10% of patients without extracraniofacial anomalies. Recent studies have not found strong correlations between anomalies associated with Goldenhar syndrome [1, 22], hypothesizing instead that the term is misused to describe a more severe form of CFM. Our results seem to support this hypothesis, as a more severe facial phenotype was associated with both more craniofacial anomalies and a history of at least one extracraniofacial anomaly.

More severe patterns of facial morphology were also seen in patients with feeding and/or breathing difficulties. Pruzansky-Kaban scores and more severe clinical presentations of CFM have been correlated to obstructive breathing and feeding difficulties in several previous studies [5, 6, 23, 24]. In the current study, significant and independent predictors of feeding and/or breathing difficulties were clefting, respiratory and/or gastrointestinal system anomalies, as well as PC34, which illustrated a morphological pattern involving mainly the upper half of the face. This pattern seems to correspond to a decreased forehead height, as well as possibly a slight downward displacement of the orbits. A previous study on syndromic patients, including patients with CFM, reported an association between decreased height of the upper half of the face and more severe obstructive sleep apnea [25]. In non-syndromic pediatric patients, no difference was found in the ratio of the upper to lower face height between patients with and without obstructive sleep apnea [26]. Interestingly, a study measuring 3D characteristics of the upper airway in patients with CFM identified smaller nasopharynx volumes in patients with obstructive breathing compared to those without [27]. This decreased nasopharynx volume could correspond to decreased upper facial height. With regard to feeding difficulties, a study on swallowing in patients with CFM identified problems in both the oral and pharyngeal phases. Inappropriate bolus formation was observed more frequently in patients with Pruzansky-Kaban 3 mandibles, while comparable Pruzansky-Kaban scores were seen in patients with difficulties in the pharyngeal phase including nasopharyngeal reflux [28]. Moreover, a small case series found impaired pharyngeal function in patients with CFM, attributing this impairment to dysplasia of pharyngeal muscles [29]. These studies suggest that feeding and breathing difficulties are not only associated with mandibular hypoplasia, but also with nasopharyngeal anatomy or dysfunction. Future studies could expand on these results by investigating the possibly decreased upper facial height found in this study, and its relation to nasopharyngeal anatomy.

Patients are often screened for extracraniofacial anomalies, as well as feeding and breathing during the first years of life [4]. We therefore undertook subgroup analyses to examine whether our main results could be extrapolated to patients under 2 years of age. We found some significant differences in FS scores, but not PC scores, although visually patients with at least one extracraniofacial anomaly seemed to have more orbital and mandibular involvement. These results tentatively suggest that morphology-based analyses may be applicable to patients in the early diagnostic stage. However, these results are limited due to the small sample size of this subgroup analysis, and larger samples are needed to confirm the applicability of these morphological assessments in young patients.

Despite including patients across three centers, our sample size was limited due to the reliance on available 3D photographs. Clinicians are encouraged to implement 3D photography into standard clinical work-up of patients, considering its accuracy, ease of use, and applicability in assessing facial morphology and treatment results comprehensively in patients with CFM [7]. Furthermore, this study was dependent on complete medical charting. The decision was made to report on all included patients and consider an anomaly not present if it was not mentioned in the chart. However, this may have led to underreporting of anomalies, especially in patients with little asymmetry in the face and isolated microtia, as screening for extracraniofacial anomalies is not always performed in this cohort. Furthermore, limited univariate analyses were performed into different types of extracraniofacial anomalies, due to the relatively small number of patients within each anomaly group.

## Conclusion

Identifying patients likely to need screening for extracraniofacial anomalies, or difficulties with breathing and feeding is important for a personalized approach to initial clinical management. The current results suggest 3D photography could be used to identify patients in need for screening by assessing facial morphology in a comprehensive way. Screening for extracraniofacial anomalies should be considered if several associated craniofacial anomalies and chin retrusion are present.

## Supplementary Information

Additional file 1: STROBE statement

Additional file 2: A visual representation of loading scores for the principal components regression model distinguishing patients with CFM from individuals without craniofacial conditions. Loading scores represent correlations between the original facial signature scores and principal component scores. Positive loadings (yellow) indicate a positive correlation with the original facial signature scores, and negative loadings (blue) represent a negative correlation. The principal component scores were generated by applying a principal components analysis on facial signature scores of a control population of individuals without craniofacial conditions, and then transforming patient scores into the same PC space

Additional file 3: All extracraniofacial anomalies

Additional file 4: Asymmetry index scores for patients with and without extracraniofacial anomalies, breathing difficulties, and feeding difficulties

Additional file 5: Facial signature scores for patients with and without extracraniofacial anomalies, breathing difficulties, and feeding difficulties

Additional file 6: Asymmetry index and facial signature scores for patients <2 years of age at the time of 3D imaging

Additional file 7: Principal component scores for patients <2 years of age at the time of 3D photography

Additional file 8: Patients under 2 years of age at the time of 3D photography with and without extracraniofacial anomalies (ECFA) (top row) or a feeding and or breathing difficulties (FBD) (bottom row) visualized using all principal components. The overlaid colors represent the corresponding facial signature scores, where orange signifies positive facial signature scores and blue negative facial signature scores

## Data Availability

Data is provided within the manuscript or supplementary information files. Additional information on the datasets used and/or analysed during the current study are available from the corresponding author on reasonable request.

## Abbreviations

CFM: Craniofacial microsomia
3D: Three-dimensional
OMENS: Orbit, Mandible, Ear, Nerve and Soft tissue classification tool
PAT-CFM: Phenotypic Assessment Tool for Craniofacial Microsomia
ECFA: Extracraniofacial anomalies
STROBE: Strengthening the Reporting of Observational studies in Epidemiology
ASI: Asymmetry index
FS: Facial signature
PC: Principal component
