# Supplementary material for: Exploring extracraniofacial anomalies in relation to facial morphology in craniofacial microsomia and microtia
Source: BMC Pediatr. 2026 Aug 7;26:1121. doi: 10.1186/s12887-026-07445-7 (PMC13732972; doi:10.1186/s12887-026-07445-7)
Supplement: Supplementary file 2 [file 12887_2026_7445_MOESM2_ESM.docx]

**Additional file 2** A visual representation of loading scores for the principal components regression model distinguishing patients with CFM from individuals without craniofacial conditions. Loading scores represent correlations between the original FS scores and principal component scores. Positive loadings (yellow) indicate a positive correlation with the original FS scores, and negative loadings (blue) represent a negative correlation. The PC scores were generated by applying a PCA on FS scores of a control population of individuals without craniofacial conditions, and then transforming patient scores into the same PC space ^1^.

| **PC13** | **PC14** | **PC15** | **PC19** | **PC20** |
| --- | --- | --- | --- | --- |
|  |  |  |  |  |

| **PC23** | **PC30** | **PC33** | **PC34** |
| --- | --- | --- | --- |
|  |  |  |  |

1. Ronde EM, de Jong GA, Nolte JW, Nienhuijs MEL, Bulstrode NW, Maal TJJ, Becking AG, Breugem CC; 3DCFMM research consortium. Three-dimensional facial morphology in patients with craniofacial microsomia and microtia. Plast Reconstr Surg. 2025 Aug 1;156(2):304-315. doi: 10.1097/PRS.0000000000011831.
