# Supplementary material for: Exploring extracraniofacial anomalies in relation to facial morphology in craniofacial microsomia and microtia
Source: BMC Pediatr. 2026 Aug 7;26:1121. doi: 10.1186/s12887-026-07445-7 (PMC13732972; doi:10.1186/s12887-026-07445-7)
Supplement: Supplementary file 3 [file 12887_2026_7445_MOESM3_ESM.docx]

**Additional file 3** All extracraniofacial anomalies

| **Cardiac anomalies** | | **Vertebral anomalies** | | **Limb anomalies** | | **Urogenital system anomalies** | | **Gastrointestinal system anomalies** | | **Respiratory system anomalies** | | **Other anomalies** | | |
| --- | --- | --- | --- | --- | --- | --- | --- | --- | --- | --- | --- | --- | --- | --- |
| *Overall* | 39 (22) | *Overall* | 39 (22) | *Overall* | 22 (12) | *Overall* | 19 (11) | *Overall* | 14 (8) | *Overall* | 10 (6) | *Overall* | 12 (7) |  |
| Ventricular septal defect | 20 (11) | Scoliosis | 23 (13) | Lower limb length discrepancy | 6 (3) | Hydronephrosis | 5 (3) | Inguinal hernia | 6 (3) | Laryngomalacia | 4 (2) | Sacral dimple | 9 (5) |  |
| Atrial septal defect | 19 (11) | Hemivertebrae | 18 (10) | Radial defect | 4 (2) | Hypospadias | 4 (2) | Esophageal atresia | 3 (2) | Tracheomalacia | 3 (2) | Vascular anomaly | 2 (1) |  |
| Patent ductus arteriosus | 10 (6) | Block vertebrae | 8 (5) | Congenital hip dysplasia | 4 (2) | Renal aplasia | 3 (2) | Umbilical hernia | 3 (2) | Bronchomalacia | 2 (1) | Other^7^ | 2 (1) |  |
| Pulmonary valve stenosis | 6 (3) | Vertebral aplasia | 8 (5) | Torticollis | 4 (2) | Undescended testis | 3 (2) | Other^5^ |  | Lung hypoplasia | 2 (1) |  |  |  |
| Aberrant subclavian artery | 3 (2) | Incomplete vertebral arch fusion | 7 (4) | Polydactyly | 2 (1) | Other^4^ | 8 (4) |  |  | Laryngeal cleft | 2 (1) |  |  |  |
| Right-sided aortic arch | 3 (2) | Cervical ribs | 7 (4) | Other^3^ | 6 (3) |  |  |  |  | Other^6^ | 2 (1) |  |  |  |
| Aortic stenosis | 3 (2) | Pectus carinatum or excavatum | 3 (2) |  |  |  |  |  |  |  |  |  |  |  |
| Fallot’s tetralogy | 2 (1) | Costovertebral fusion | 2 (1) |  |  |  |  |  |  |  |  |  |  |  |
| Anomalous venous return | 2 (1) | Unspecified | 3 (2) |  |  |  |  |  |  |  |  |  |  |  |
| Unspecified | 1 (1) | Other^2^ | 6 (3) |  |  |  |  |  |  |  |  |  |  |  |
| Other^1^ | 3 (2) |  |  |  |  |  |  |  |  |  |  |  |  |  |
| ^1^ Bicuspid aortic valve (1), mesocardia (1), aorta insufficiency (1). | | | | | | | | | | | | | | |
| ^2^ Unsegmented bar (1), facet joint ankylosis (1), costal fusion (1), extra vertebra (1), extra pedicle (1), shape malformation NS (1). | | | | | | | | | | | | | | |
| ^3^ Smaller foot (1), ACL insufficiency (1), trigger thumb (1), micromelia upper limb (1), ulnar deviation hand (1), club foot (1); sternocleidomastoid muscle hypoplasia (1) | | | | | | | | | | | | | | |
| ^4^ Retractile testis (1), phimosis (1), mega ureter (1), duplicate system (1), kidney stones (1), malpositioned kidney (1), utero-vaginal aplasia (1) | | | | | | | | | | | | | | |
| ^5^ Anal atresia (1), tracheoesophageal fistula (1) | | | | | | | | | | | | | | |
| ^6^ Choanal atresia (1), larynx deviation (1) | | | | | | | | | | | | | | |
| ^7^ Thymus hyperplasia (1), Café au Lait spots | | | | | | | | | | | | | | |
