# Supplementary material for: Exploring extracraniofacial anomalies in relation to facial morphology in craniofacial microsomia and microtia
Source: BMC Pediatr. 2026 Aug 7;26:1121. doi: 10.1186/s12887-026-07445-7 (PMC13732972; doi:10.1186/s12887-026-07445-7)
Supplement: Supplementary file 4 [file 12887_2026_7445_MOESM4_ESM.docx]

**Additional file 4** ASI scores for patients with and without extracraniofacial anomalies, breathing difficulties and feeding difficulties.

| **Regions^1^** |  | 1   | 2   | 3   | 4   | 5   | 6   | 7   | 8   | 9   | 10   | 11   |  |
| --- | --- | --- | --- | --- | --- | --- | --- | --- | --- | --- | --- | --- | --- |
| *Cardiac anomalies* | Yes, n=36 | 1.78** | 1.22* | 1.82** | 1.42* | 0.94* | 2.06* | 1.33* | 1.82* | 2.23* | 1.30* | 1.35* |  |
|  |  | [1.34-2.25] | [0.71-1.91] | [1.45-2.41] | [0.72-1.81] | [0.69-1.63] | [1.34-2.73] | [1.08-1.68] | [1.16-2.45] | [1.52-3.02] | [1.01-1.64] | [1.14-1.66] |  |
|  | No, n=124 | 1.28 | 0.91 | 1.33 | 0.87 | 0.75 | 1.45 | 1.12 | 1.24 | 1.59 | 1.00 | 1.14 |  |
|  |  | [0.95-1.94] | [0.65-1.20] | [0.97-2.09] | [0.65-1.26] | [0.55-1.06] | [0.92-2.44] | [0.80-1.47] | [0.76-1.95] | [0.97-2.72] | [0.63-1.43] | [0.78-1.51] |  |
| *Vertebral anomalies* | Yes, n=33 | 1.79◦ | 1.03 | 1.95◦ | 0.94 | 1.00* | 2.30 | 1.30 | 1.68◦ | 2.33 | 1.11 | 1.35 |  |
|  |  | [1.06-2.41] | [0.70-1.58] | [1.08-2.52] | [0.76-1.57] | [0.71-1.40] | [1.10-2.95] | [0.94-1.57] | [1.21-2.47] | [1.11-3.12] | [0.75-1.57] | [0.89-1.57] |  |
|  | No, n=127 | 1.35 | 0.93 | 1.44 | 0.89 | 0.75 | 1.53 | 1.15 | 1.32 | 1.72 | 1.05 | 1.19 |  |
|  |  | [0.97-1.90] | [0.65-1.22] | [1.01-2.05] | [0.63-1.48] | [0.56-1.05] | [0.98-2.41] | [0.81-1.48] | [0.83-1.97] | [1.01-2.69] | [0.67-1.47] | [0.78-1.51] |  |
| *Limb anomalies* | Yes, n=18 | 1.71 | 1.01 | 1.58 | 1.01 | 0.77 | 1.48 | 1.33 | 1.39 | 1.71 | 1.10 | 1.48◦ |  |
|  |  | [1.08-2.23] | [0.60-1.70] | [1.09-2.28] | [0.68-1.87] | [0.62-1.28] | [0.90-2.81] | [0.97-1.77] | [0.78-2.45] | [0.93-3.11] | [0.81-1.58] | [1.03-1.76] |  |
|  | No, n=142 | 1.37 | 0.93 | 1.46 | 0.92 | 0.80 | 1.57 | 1.15 | 1.40 | 1.75 | 1.06 | 1.19 |  |
|  |  | [1.00-1.97] | [0.66-1.26] | [1.04-2.11] | [0.65-1.48] | [0.56-1.13] | [1.00-2.47] | [0.83-1.48] | [0.88-2.06] | [1.06-2.76] | [0.69-1.49] | [0.82-1.50] |  |
| *Urogenital system anomalies* | Yes, n=16 | 1.38 | 0.98 | 1.49 | 1.07 | 0.82 | 1.74 | 1.03 | 1.41 | 1.96 | 0.95 | 1.01 |  |
|  |  | [0.99-1.99] | [0.68-1.23] | [1.00-2.08] | [0.61-1.48] | [0.50-1.31] | [0.94-2.46] | [0.84-1.33] | [0.96-2.02] | [0.84-2.60] | [0.73-1.33] | [0.91-1.46] |  |
|  | No, n=144 | 1.39 | 0.93 | 1.47 | 0.90 | 0.78 | 1.56 | 1.20 | 1.40 | 1.71 | 1.07 | 1.21 |  |
|  |  | [1.01-2.07] | [0.66-1.27] | [1.06-2.15] | [0.66-1.51] | [0.58-1.15] | [1.02-2.50] | [0.87-1.53] | [0.83-2.16] | [1.06-2.82] | [0.70-1.51] | [0.82-1.54] |  |
| *Gastrointestinal system anomalies* | Yes, n=13 | 1.78 | 1.29 | 1.67 | 1.24 | 0.96 | 1.90 | 1.32 | 1.51 | 2.17 | 1.39 | 1.32 |  |
|  |  | [1.34-2.06] | [0.70-1.60] | [1.46-2.15] | [0.69-1.86] | [0.61-1.26] | [1.27-2.79] | [1.07-1.71] | [1.11-2.55] | [1.25-2.76] | [0.71-1.73] | [1.22-1.65] |  |
|  | No, n=147 | 1.36 | 0.93 | 1.44 | 0.91 | 0.78 | 1.56 | 1.18 | 1.38 | 1.72 | 1.05 | 1.19 |  |
|  |  | [1.00-2.08] | [0.65-1.23] | [1.02-2.12] | [0.65-1.49] | [0.56-1.15] | [0.98-2.49] | [0.82-1.48] | [0.83-2.12] | [0.99-2.78] | [0.70-1.48] | [0.79-1.53] |  |
| *Respiratory* *system anomalies* | Yes, n=8 | 2.09* | 1.27◦ | 2.22* | 1.50◦ | 0.91 | 2.78* | 1.56 | 2.07◦ | 3.17* | 1.50◦ | 1.42 |  |
|  |  | [1.79-2.29] | [1.07-1.42] | [1.93-2.41] | [1.23-1.67] | [0.58-1.27] | [2.16-2.97] | [1.04-1.64] | [1.51-2.52] | [2.46-3.45] | [1.33-1.85] | [0.97-1.61] |  |
|  | No, n=152 | 1.37 | 0.92 | 1.44 | 0.89 | 0.79 | 1.54 | 1.17 | 1.37 | 1.71 | 1.06 | 1.20 |  |
|  |  | [1.00-2.02] | [0.65-1.26] | [1.03-2.09] | [0.64-1.49] | [0.57-1.15] | [0.98-2.45] | [0.84-1.48] | [0.83-2.04] | [1.01-2.72] | [0.68-1.44] | [0.82-1.54] |  |
| *Other ECFA* | Yes, n=11 | 2.15* | 1.21◦ | 2.31* | 1.10◦ | 0.90◦ | 2.79* | 1.43 | 2.23* | 3.26* | 1.58 | 1.40 |  |
|  |  | [1.44-2.74] | [0.93-1.49] | [1.50-2.97] | [0.89-1.78] | [0.85-1.26] | [1.63-3.72] | [1.05-1.69] | [1.43-3.46] | [1.83-4.13] | [0.86-1.98] | [1.11-1.49] |  |
|  | No, n=149 | 1.36 | 0.93 | 1.45 | 0.89 | 0.77 | 1.53 | 1.15 | 1.32 | 1.71 | 1.06 | 1.19 |  |
|  |  | [1.00-1.94] | [0.65-1.26] | [1.03-2.09] | [0.63-1.49] | [0.56-1.14] | [0.97-2.44] | [0.83-1.49] | [0.83-2.01] | [1.01-2.73] | [0.70-1.45] | [0.80-1.54] |  |
| *Breathing difficulties* | Yes, n=23 | 1.86 | 1.32** | 1.93 | 1.40* | 1.05* | 1.93 | 1.21 | 1.97 | 2.17 | 1.07 | 1.32 |  |
|  |  | [1.26-2.16] | [0.91-1.67] | [1.22-2.22] | [0.99-1.86] | [0.69-1.62] | [1.31-2.74] | [0.93-1.60] | [1.09-2.38] | [1.44-2.90] | [0.69-1.50] | [0.88-1.73] |  |
|  | No, n=137 | 1.36 | 0.91 | 1.44 | 0.88 | 0.77 | 1.52 | 1.18 | 1.32 | 1.72 | 1.06 | 1.19 |  |
|  |  | [0.97-2.01] | [0.65-1.20] | [1.01-2.09] | [0.64-1.42] | [0.56-1.05] | [0.97-2.48] | [0.83-1.48] | [0.83-1.99] | [0.98-2.73] | [0.73-1.49] | [0.82-1.54] |  |
| *Feeding difficulties* | Yes, n=150 | 1.96** | 1.21** | 2.09** | 1.51*** | 1.00* | 2.43* | 1.40* | 1.95* | 2.58* | 1.31* | 1.36◦ |  |
|  |  | [1.39-2.30] | [0.85-1.96] | [1.46-2.40] | [0.85-1.95] | [0.68-1.63] | [1.29-2.82] | [1.06-1.73] | [1.19-2.54] | [1.35-3.27] | [0.87-1.85] | [1.06-1.61] |  |
|  | No, n=29 | 1.30 | 0.91 | 1.35 | 0.88 | 0.75 | 1.49 | 1.15 | 1.26 | 1.63 | 1.04 | 1.18 |  |
|  |  | [0.97-1.90] | [0.65-1.21] | [1.00-2.05] | [0.62-1.26] | [0.56-1.08] | [0.95-2.40] | [0.81-1.45] | [0.82-2.00] | [0.99-2.61] | [0.63-1.43] | [0.80-1.50] |  |
| ECFA: extracraniofacial anomalies | | | | | | | | | | | | | |
| ^1^Median (interquartile range) root mean square | | | | | | | | | | | | | |
| ***p<0.001, **p<0.01, *p<0.05, ◦ p≤0.1, Mann Whitney U test. | | | | | | | | | | | | | |
