# Supplementary material for: Exploring extracraniofacial anomalies in relation to facial morphology in craniofacial microsomia and microtia
Source: BMC Pediatr. 2026 Aug 7;26:1121. doi: 10.1186/s12887-026-07445-7 (PMC13732972; doi:10.1186/s12887-026-07445-7)
Supplement: Supplementary file 5 [file 12887_2026_7445_MOESM5_ESM.docx]

**Additional file 5** FS scores for patients with and without extracraniofacial anomalies, breathing difficulties and feeding difficulties.

| Regions^1^ |  | 1   | 2   | 3   | 4   | 5   | 6   | 7   | 8   | 9   | 10   | 11   |  |
| --- | --- | --- | --- | --- | --- | --- | --- | --- | --- | --- | --- | --- | --- |
| *Cardiac anomalies* | Yes, n=41 | 1.52* | 1.32** | 1.58◦ | 1.29 | 1.29 | 1.73** | 1.34* | 1.34 | 1.64** | 1.21 | 1.30 |  |
|  |  | [1.11-2.04] | [1.07-1.71] | [1.14-1.96] | [0.90-1.58] | [1.03-1.81] | [1.20-2.14] | [1.07-1.65] | [1.12-1.87] | [1.24-2.42] | [0.82-1.58] | [0.99-1.61] |  |
|  | No, n=138 | 1.33 | 1.12 | 1.36 | 1.14 | 1.04 | 1.37 | 1.28 | 1.11 | 1.44 | 1.14 | 1.20 |  |
|  |  | [1.14-1.60] | [0.89-1.42] | [1.14-1.64] | [0.86-1.54] | [0.84-1.39] | [1.09-1.82] | [0.98-1.56] | [0.90-1.56] | [1.11-1.92] | [0.84-1.61] | [0.96-1.45] |  |
| *Vertebral anomalies* | Yes, n=39 | 1.44 | 1.33 | 1.51 | 1.24 | 1.29 | 1.48 | 1.35 | 1.29 | 1.57 | 1.19 | 1.34* |  |
|  |  | [1.22-1.69] | [0.92-1.62] | [1.24-1.72] | [0.85-1.69] | [0.85-1.75] | [1.22-1.91] | [1.14-1.56] | [0.99-1.86] | [1.24-2.08] | [0.96-1.54] | [1.13-1.56] |  |
|  | No, n=140 | 1.34 | 1.15 | 1.39 | 1.15 | 1.11 | 1.41 | 1.28 | 1.19 | 1.43 | 1.16 | 1.18 |  |
|  |  | [1.10-1.67] | [0.94-1.44] | [1.12-1.73] | [0.87-1.53] | [0.87-1.39] | [1.09-1.86] | [0.94-1.59] | [0.92-1.58] | [1.13-2.04] | [0.82-1.66] | [0.93-1.45] |  |
| *Limb anomalies* | Yes, n=22 | 1.36 | 1.13 | 1.41 | 1.09 | 1.13 | 1.32 | 1.34 | 1.21 | 1.36 | 1.24 | 1.28 |  |
|  |  | [1.09-1.69] | [0.87-1.68] | [1.10-1.70] | [0.76-1.81] | [0.82-1.50] | [0.99-1.87] | [1.13-1.56] | [0.84-1.66] | [1.11-1.66] | [0.99-1.78] | [1.07-1.47] |  |
|  | No, n=157 | 1.37 | 1.18 | 1.42 | 1.16 | 1.12 | 1.43 | 1.29 | 1.20 | 1.51 | 1.14 | 1.20 |  |
|  |  | [1.15-1.66] | [0.94-1.46] | [1.14-1.73] | [0.88-1.53] | [0.87-1.45] | [1.10-1.90] | [0.96-1.59] | [0.94-1.62] | [1.15-2.10] | [0.82-1.60] | [0.96-1.47] |  |
| *Urogenital system anomalies* | Yes, n=18 | 1.25◦ | 1.19 | 1.28◦ | 1.06 | 1.09 | 1.17* | 1.31** | 0.94* | 1.24 | 1.17 | 1.25 |  |
|  |  | [0.99-1.47] | [0.88-1.37] | [0.98-1.51] | [0.79-1.37] | [0.92-1.45] | [1.00-1.33] | [0.92-1.57] | [0.82-1.16] | [0.95-1.58] | [0.82-1.31] | [0.95-1.62] |  |
|  | No, n=160 | 1.42 | 1.17 | 1.44 | 1.17 | 1.13 | 1.46 | 1.29 | 1.23 | 1.53 | 1.17 | 1.21 |  |
|  |  | [1.16-1.69] | [0.94-1.48] | [1.15-1.75] | [0.87-1.58] | [0.86-1.45] | [1.10-1.92] | [0.99-1.59] | [0.95-1.66] | [1.16-2.10] | [0.83-1.63] | [0.97-1.45] |  |
| *Gastrointestinal system anomalies* | Yes, n=14 | 1.62 | 1.43◦ | 1.63 | 1.30 | 1.23 | 1.86* | 1.35 | 1.77* | 1.71 | 1.11 | 1.43 |  |
|  |  | [1.30-2.01] | [1.08-1.85] | [1.35-1.96] | [1.02-1.84] | [1.05-1.93] | [1.37-2.24] | [1.18-1.52] | [1.12-2.07] | [1.56-2.56] | [0.87-1.38] | [1.04-1.55] |  |
|  | No, n=165 | 1.35 | 1.17 | 1.39 | 1.15 | 1.11 | 1.40 | 1.29 | 1.19 | 1.44 | 1.17 | 1.20 |  |
|  |  | [1.11-1.65] | [0.92-1.45] | [1.14-1.70] | [0.86-1.53] | [0.86-1.41] | [1.09-1.84] | [0.97-1.59] | [0.92-1.57] | [1.13-2.02] | [0.83-1.61] | [0.96-1.45] |  |
| *Respiratory* *system anomalies* | Yes, n=10 | 1.51 | 1.13 | 1.60 | 1.08 | 1.11 | 1.69 | 1.34 | 1.38 | 1.89 | 1.37 | 1.19 |  |
|  |  | [1.15-1.71] | [0.92-1.50] | [1.13-1.81] | [0.84-1.55] | [0.86-1.46] | [1.16-2.12] | [1.17-1.59] | [0.93-1.89] | [1.35-2.22] | [1.19-1.71] | [1.01-1.48] |  |
|  | No, n=169 | 1.35 | 1.18 | 1.41 | 1.16 | 1.13 | 1.42 | 1.29 | 1.20 | 1.45 | 1.14 | 1.22 |  |
|  |  | [1.13-1.66] | [0.94-1.46] | [1.14-1.73] | [0.87-1.54] | [0.86-1.45] | [1.09-1.87] | [0.97-1.58] | [0.93-1.62] | [1.13-2.02] | [0.83-1.60] | [0.96-1.47] |  |
| *Other ECFA* | Yes, n=12 | 1.72* | 1.71* | 1.71* | 1.79* | 1.55* | 1.85* | 1.40 | 1.54◦ | 1.99* | 1.35 | 1.42* |  |
|  |  | [1.40-1.99] | [1.25-1.93] | [1.46-1.93] | [0.92-1.99] | [1.09-2.02] | [1.50-2.25] | [1.30-1.60] | [1.07-2.37] | [1.63-2.22] | [1.10-1.57] | [1.27-1.63] |  |
|  | No, n=167 | 1.35 | 1.16 | 1.39 | 1.15 | 1.12 | 1.40 | 1.28 | 1.20 | 1.44 | 1.15 | 1.20 |  |
|  |  | [1.10-1.63] | [0.93-1.44] | [1.11-1.69] | [0.86-1.52] | [0.86-1.41] | [1.08-1.85] | [0.97-1.56] | [0.93-1.58] | [1.12-1.99] | [0.82-1.62] | [0.96-1.45] |  |
| *Breathing difficulties* | Yes, n=29 | 1.56◦ | 1.40** | 1.57 | 1.58** | 1.32* | 1.72* | 1.36 | 1.44* | 1.66* | 1.14 | 1.41◦ |  |
|  |  | [1.17-2.06] | [1.13-1.80] | [1.19-1.98] | [1.05-1.84] | [1.03-1.77] | [1.12-2.31] | [1.12-1.59] | [1.09-1.90] | [1.24-2.70] | [0.91-1.60] | [1.06-1.58] |  |
|  | No, n=150 | 1.35 | 1.14 | 1.39 | 1.14 | 1.09 | 1.37 | 1.28 | 1.17 | 1.43 | 1.17 | 1.20 |  |
|  |  | [1.11-1.62] | [0.91-1.43] | [1.13-1.69] | [0.86-1.48] | [0.86-1.41] | [1.09-1.83] | [0.97-1.57] | [0.91-1.57] | [1.12-1.93] | [0.82-1.61] | [0.96-1.45] |  |
| *Feeding difficulties* | Yes, n=35 | 1.72*** | 1.56*** | 1.73 | 1.58*** | 1.45*** | 1.87*** | 1.42** | 1.55** | 1.74** | 1.19* | 1.39** |  |
|  |  | [1.43-2.11] | [1.13-1.94] | [1.49-2.02] | [1.11-1.88] | [1.13-1.97] | [1.39-2.24] | [1.17-1.82] | [1.07-2.07] | [1.47-2.49] | [0.99-2.15] | [1.18-1.77] |  |
|  | No, n=144 | 1.31 | 1.12 | 1.33 | 1.08 | 1.04 | 1.33 | 1.23 | 1.13 | 1.39 | 1.16 | 1.18 |  |
|  |  | [1.10-1.56] | [0.90-1.40] | [1.12-1.59] | [0.80-1.46] | [0.84-1.36] | [1.06-1.80] | [0.96-1.51] | [0.90-1.54] | [1.08-1.92] | [0.81-1.55] | [0.93-1.43] |  |
| ECFA: extracraniofacial anomalies | | | | | | | | | | | | | |
| ^1^Median (interquartile range) root mean square | | | | | | | | | | | | | |
| ***p<0.001, **p<0.01, *p<0.05, ◦ p≤0.1, Mann Whitney U test. | | | | | | | | | | | | | |
