# Supplementary material for: Exploring extracraniofacial anomalies in relation to facial morphology in craniofacial microsomia and microtia
Source: BMC Pediatr. 2026 Aug 7;26:1121. doi: 10.1186/s12887-026-07445-7 (PMC13732972; doi:10.1186/s12887-026-07445-7)
Supplement: Supplementary file 6 [file 12887_2026_7445_MOESM6_ESM.docx]

**Additional file 6** ASI and FS scores for patients <2 years of age at the time of 3D imaging.

| Regions^1^ |  |  | 1 | 2 | 3 | 4 | 5 | 6 | 7 | 8 | 9 | 10 | 11 |
| --- | --- | --- | --- | --- | --- | --- | --- | --- | --- | --- | --- | --- | --- |
|  |  |  |  |  |  |  |  |  |  |  |  |  |  |
| **ASI** | *Any ECFA* | **Yes,** n=7 | 1.33  (0.67-2.69) | 1.38  (0.41-2.55) | 1.44  (0.68-2.88) | 0.94  (0.32-2.92) | 1.27  (0.46-2.27) | 1.44  (0.72-3.58) | 1.43  (0.64-1.83) | 1.45  (0.57-3.46) | 1.56  (0.82-3.66) | 1.61  (0.38-2.02) | 1.19  (0.75-1.94) |
|  |  | **No,** n=4 | 0.89  (0.71-1.24) | 0.70  (0.47-0.91) | 0.91  (0.69-1.36) | 0.70  (0.55-1.27) | 0.53  (0.41-0.87) | 1.12  (0.79-1.56) | 0.59  (0.49-1.07) | 0.87  (0.79-1.54) | 1.30  (0.71-1.57) | 0.71  (0.48-1.15) | 0.55  (0.38-1.02) |
|  | *History of FBD* | **Yes,** n=4 | 1.50  (0.81-2.41) | 1.51  (0.60-2.55) | 1.37  (0.85-2.56) | 1.39  (0.71-2.92) | 1.54  (0.51-2.27) | 1.32  (0.99-3.09) | 1.31  (0.62-1.83) | 1.21  (0.79-3.05) | 1.37  (1.13-3.12) | 1.15  (0.64-1.91) | 1.30  (0.61-1.94) |
|  |  | **No,** n=7 | 1.23  (0.67-2.69) | 0.65  (0.41-1.52) | 1.35  (0.68-2.88) | 0.69  (0.32-1.83) | 0.56  (0.41-1.27) | 1.44  (0.72-3.58) | 0.94  (0.49-1.67) | 1.45  (0.57-3.46) | 1.48  (0.71-3.66) | 0.78  (0.38-2.02) | 1.02  (0.38-1.45) |
| **FS** | *Any ECFA* | **Yes,** n=7 | 1.73  (1.07 -2.15) | 1.42  (1.02 -2.68) | 1.85  (0.95 -2.23) | 1.34  (0.79 -1.83) | 1.34  (0.97 -3.12) | 2.06  (0.93 -2.46) | 1.32  (0.88 -2.41) | 1.98  (1.02 -2.57) | 1.89  (0.84 -2.77) | 1.36  (0.68 -3.11) | 1.36  (0.96 -2.18) |
|  |  | **No,** n=5 | 1.45  (1.09-1.70) | 1.38  (1.11-1.99) | 1.47  (1.02-1.63) | 1.54  (1.09-2.52) | 1.20  (0.65-1.98) | 1.56  (1.09-1.94) | 0.91  (0.84- 1.60) | 1.53  (0.97-2.17) | 1.74  (1.18 -2.02) | 0.79  (0.43- 2.41) | 0.87  (0.77- 1.35) |
|  | *History of FBD* | **Yes,** n=4 | 2.07*  (1.70-2.15) | 1.89*  (1.42-2.68) | 2.05*  (1.63-2.23) | 1.81  (1.29-2.00) | 1.89*  (1.50-3.12) | 2.08*  (1.94-2.46) | 1.74◦  (1.15-2.41) | 2.12*  (1.98-2.57) | 1.90  (1.64-2.77) | 1.59  (0.66-3.11) | 1.64*  (1.35-2.18) |
|  |  | **No,** n=8 | 1.36  (1.07-1.73) | 1.14  (1.02 -1.68) | 1.38  (0.95 -1.85) | 1.42  (0.79 -2.52) | 1.12  (0.65 -1.34) | 1.49  (0.93 -2.19) | 0.95  (0.84 -1.60) | 1.37  (0.97 -2.28) | 1.58  (0.84 -2.12) | 1.00  (0.43 -2.41) | 0.97  (0.77 -1.36) |
| ECFA: extracraniofacial anomalies; FBD: feeding and/or breathing difficulties | | | | | | | | | | | | | |
| ^1^Median (interquartile range) root mean square | | | | | | | | | | | | | |
| ***p<0.001, **p<0.01, *p<0.05, ◦ p≤0.1, Mann Whitney U test. Adjusted for multiple testing. | | | | | | | | | | | | | |
