# Supplementary material for: Exploring extracraniofacial anomalies in relation to facial morphology in craniofacial microsomia and microtia
Source: BMC Pediatr. 2026 Aug 7;26:1121. doi: 10.1186/s12887-026-07445-7 (PMC13732972; doi:10.1186/s12887-026-07445-7)
Supplement: Supplementary file 7 [file 12887_2026_7445_MOESM7_ESM.docx]

**Additional file 7** PC scores for patients <2 years of age at the time of 3D imaging

| **Type of variable** | **Extracraniofacial anomalies** | | | **Feeding and/or breathing difficulties** | | |
| --- | --- | --- | --- | --- | --- | --- |
|  | **No,** n=5 | **Yes,** n=7 | *p-value^2^* | **No,** n=8 | **Yes,** n=4 | *p-value^2^* |
| **Principal components** |  |  |  |  |  |  |
| PC13 | -9.95 (-43.40--3.83) | -15.43 (-25.73-6.76) | 0.7 | -14.22 (-43.40-6.76) | -14.58 (-25.73--3.83) | >0.9 |
| PC14 | -1.82 (-34.82-10.14) | -11.35 (-59.48-1.64) | 0.7 | -7.08 (-59.48-10.14) | -5.48 (-48.95-1.64) | >0.9 |
| PC15 | -3.59 (-11.33-31.41) | -10.27 (-23.65-18.28) | 0.7 | -6.09 (-23.65-8.52) | 4.01 (-19.36-31.41) | >0.9 |
| PC19 | -3.56 (-18.83-7.37) | -18.55 (-30.36-0.55) | 0.7 | -8.87 (-30.36-0.84) | -9.00 (-29.43-7.37) | >0.9 |
| PC20 | -4.49 (-19.29--1.14) | -22.92 (-41.43-0.95) | 0.7 | -11.79 (-39.36--1.14) | -9.76 (-41.43-0.95) | >0.9 |
| PC23 | -3.03 (-12.74-1.58) | 1.73 (-19.24-17.93) | 0.7 | -2.57 (-12.74-17.93) | -0.65 (-19.24-11.14) | >0.9 |
| PC30 | 14.69 (4.81-17.34) | 14.76 (4.55-19.54) | 0.8 | 14.73 (4.55-17.34) | 16.14 (4.81-19.54) | >0.9 |
| PC33 | 7.78 (-1.71-13.95) | 1.02 (-4.53-15.51) | 0.7 | 2.26 (-4.53-13.95) | 1.30 (0.78-15.51) | >0.9 |
| PC34 | 0.15 (-8.91-6.42) | 0.47 (-8.50-5.42) | 0.7 | 0.46 (-8.50-6.42) | 0.46 (-8.91-5.42) | >0.9 |
| ^1^Median (range) or Number (%) | | | | | |  |
| ^2^Mann Whitney U test; Fisher’s exact test. Adjusted for multiple testing. | | | | | |  |
