# Supplementary material for: Exploring extracraniofacial anomalies in relation to facial morphology in craniofacial microsomia and microtia
Source: BMC Pediatr. 2026 Aug 7;26:1121. doi: 10.1186/s12887-026-07445-7 (PMC13732972; doi:10.1186/s12887-026-07445-7)
Supplement: Supplementary file 8 [file 12887_2026_7445_MOESM8_ESM.docx]

**Additional file 8** Patients <2 years of age at the time of 3D imaging with and without extracraniofacial anomalies (ECFA) or a feeding and or breathing difficulties (FBD) visualized using all principal components. The overlaid colors represent the corresponding FS scores, where orange signifies positive FS scores and blue negative FS scores.
